# Inverting the Body in Chloroform Poisoning

**Published:** 1875-02

**Authors:** 


### INVERTING THE BODY IN CHLOROFORM POISONING.

If dentists and doctors are to go on using chloroform as an anæsthetic, they ought to be familiar with what is known in Europe as "Nelaton's method" of dealing with cases of narcosis occurring from its administration. This treatment was the subject of two communications at the late meeting of the

British Medical Association at Norwich, Eng., where it excited much interest. The first of these was so graphic a description of the method that we reprint the full report of it given in *The Doctor:*—

Dr. Marion Sims thus related a case in which Nelaton was present: "In the midst of the operation, the chloroformist, Dr. Campbell, said, 'Stop! stop! No pulse, no breathing!' and, looking to M. Nelaton, he said, 'Tete en bas, n'est-ce pas?' Nelaton replied, 'Certainly; there is nothing else to do.' Immediately the body was inverted, the head hanging down, while the heels were raised high in the air by Dr. Johnston, the legs resting, one on each of his shoulders. Dr. Campbell supported the thorax. Mr. Herbert was sent to an adjoining room for a spoon, with the handle of which the jaws were held open, and I handed M. Nelaton a tenaculum, which he hooked into the tongue, and gave in charge to Mr. Herbert; while to Dr. Beylard was assigned the duty of making efforts at artificial respiration, by pressure alternately on the thorax and abdomen. M. Nelaton ordered and overlooked every movement, while I stood aloof and watched the proceedings with, of course, the most intense anxiety. They held the patient in this inverted position for a long time before there was any manifestation of returning life. Dr. Campbell, in his report, says it was fifteen minutes, and that it seemed an age. My notes of the case, written a few hours afterward, make it twenty minutes. Be this as it may, the time was so long that I thought it useless to make any further efforts, and I said, "Gentlemen, she is certainly dead, and you might as well let her alone." But the great and good Nelaton never lost hope, and by his quiet, cool, brave manner he seemed to infuse his spirit into his aids. At last there was a feeble inspiration, and after a long time another, and by and by another; and then the breathing became pretty regular, and Dr. Campbell said, 'The pulse returns, thank God; she will soon be all right again.' Dr. Beylard, who always sees the cheerful side of everything in life, was disposed to laugh at the fear I manifested for the safety of our patient. I must confess that never before or since have I felt such a grave responsibility.

When the pulse and respiration were well reestablished, M. Nelaton ordered the patient to be laid upon the table. This was done gently. But what was our horror, when at the moment the body was placed horizontally, the pulse and breathing instantly ceased. Quick as thought the body was again inverted, the head downward and the feet over Dr. Johnston's shoulders, and the same manœuvres as before were put in execution. Dr. Campbell thinks it did not take such a long time to re-establish the action of the lungs and heart as in the first instance. It may have lacked a few seconds of the time; but it seemed to me to be quite as long. For the same tedious, painful, protracted, and anxious methods were made as before, and she seemed, if possible, more dead than before; but thanks to the brave men who had her in charge, feeble signs of returning life eventually made their appearance. Respiration was at first irregular, and at long intervals; soon it became more regular, and the pulse could then be counted; but it was very feeble, and would intermit. I began again to be hopeful, and even dared to think that at last there was an end of the dreadful suspense, when they laid her horizontally on the table again, saying, 'She is all right this time.' To witness two such painful scenes of danger to a young and valuable life, and to experience such agony of anxiety, produced a tension of heart, and mind, and soul, that can not be imagined. What, then must have been our dismay or feeling of despair when, incredible as it may seem, the moment the body was laid in the horizontal position again, the respiration ceased a third time, the pulse was gone, and she looked the perfect picture of death! Then I gave up all as lost, for I thought that the blood was so poisoned, so charged with chloroform, that it was no longer able to sustain life. But Nelaton and Campbell and Johnston and Beylard and Herbert, by a consentaneous effort, quickly inverted the body a third time, thus throwing all the blood possible to the brain, and again they began their efforts at artificial respiration. It seemed to me that she would never breathe again; but at last there was a spasmodic gasp, and after a long while, there was another effort at inspiration, and after another long interval, there was a third; they were 'far between;' then we watched and waited and wondered if there would ever be a fourth; at length it came, and more profoundly, and there was a long yawn, and the respiration became tolerably regular. Soon Dr. Beylard says, 'I feel the pulse again, but it is very weak.' Nelaton, after some moments, ejaculates, 'The color of the tongue and lips is more natural.' Campbell says, 'The vomiting is favorable: see, she moves her hands; she is pushing against me.' But I was by no means sure that these movements were not merely signs of the last death-struggle, and so I expressed myself. Presently Dr. Johnston said, 'See here, doctor; see how she kicks; she is coming round again;' and very soon they all said, 'She is safe at last.' I replied, 'For Heaven's sake keep her safe; I beg you not to put her on the table again till she is conscious.' This was the first and only suggestion I made during all these anxious moments, and it was acted upon, for she was held in the vertical position till she, in a manner, recovered semi-consciousness, opened her eyes, looked wildly around, and asked what was the matter. She was then, and not till then, laid on the table, and all present felt quite as solemn and as thankful as I did; and we all in turn grasped Nelaton's hand, and thanked him for having saved the life of this lovely woman."

The other communication on the subject was from Sir John Rose Cormack, who described a case in which the method had proved successful, although the patient remained for a much longer time in a precarious condition.

